# *Dehydration-Responsive Element Binding Protein 1C*, *1E*, and *1G* Promote Stress Tolerance to Chilling, Heat, Drought, and Salt in Rice

**DOI:** 10.3389/fpls.2022.851731

**Published:** 2022-05-24

**Authors:** Huanhuan Wang, Shan Lu, Xiangyu Guan, Yuan Jiang, Bin Wang, Jian Hua, Baohong Zou

**Affiliations:** ^1^The State Key Laboratory of Crop Genetics and Germplasm Enhancement, Nanjing Agricultural University, Nanjing, China; ^2^Plant Biology Section, School of Integrated Plant Science, Cornell University, Ithaca, NY, United States; ^3^Department of Electrical and Electronic Engineering, Guilin University of Technology, Nanning, China

**Keywords:** *OsDREB1*, chilling tolerance, cold acclimation, abiotic stress, ROS, cell death, rice

## Abstract

The *dehydration-responsive element binding protein 1* (*DREB1*)/*C-repeat-binding factor* (*CBF*) genes are key regulators of cold acclimation and freezing tolerance in the chilling tolerant *Arabidopsis thaliana*. Here, we investigated the function of three members of the 10 rice *DREB1* genes, *OsDREB1C*, *E*, and *G*, in the chilling sensitive rice plants. Their loss of function (LOF) mutants were each more chilling susceptible compared to the wild type, and the LOF mutants of all three genes, *dreb1ceg*, were more chilling susceptible than any of the single mutants. Strikingly, these mutants were capable of cold acclimation, indicating that these rice *DREB1* genes are important for basal chilling tolerance but not cold acclimation. Transcriptome and physiology analyses suggest that the *OsDREB1C*/*E*/*G* genes are involved in reactive oxygen species (ROS) scavenging and cell death regulation under chilling. Furthermore, these three rice *DREB1* genes are found to promote tolerance to other abiotic stresses: the *OsDREB1C*/*E*/*G* genes are positive regulators of heat tolerance, *OsDREB1C* and *OsDREB1G* are positive regulators of salt tolerance, and *OsDREB1G* is a positive regulator of drought tolerance. These findings expand our knowledge of the roles of DREB1 proteins in plants, enhance our mechanistic understanding of abiotic stress tolerance and will facilitate the generation of stress-tolerant crop plants.

## Introduction

Cold stresses, both freezing and chilling (above freezing), are a major threat to crop production worldwide (Pearce, 2001; Zhang et al., 2019). Rice (*Oryza sativa*), with a tropical and temperate origin, is susceptible to cold stress at all developmental stages (Zhang et al., 2014). A temperature lower than 15°C causes drastic physiological changes and inhibits the growth and development of rice (Jacobs and Pearson, 1999; Aghaee et al., 2011). Chilling stress results in poor germination, stunted seedlings, yellowing or withering of leaves, and reduced tillering in rice (Yadav, 2010). Chilling induces accumulation of reactive oxygen species (ROS) which serves as both a signaling molecule of cold response and a damaging molecule to the cell (Hasanuzzaman et al., 2013; Devireddy et al., 2021). Excess ROS is harmful to the stability of cell membrane and proteins and leads to growth inhibition or cell death (Mittler, 2002; Mhamdi and Van Breusegem, 2018). ROS-scavenging has an important role in protecting plants against chilling stress, and a correlation of ROS scavenging and chilling tolerance has been reported (Fang et al., 2020; Ge et al., 2020).

Plants have multiple mechanisms to promote tolerance to cold stresses. The most extensively studied process is cold acclimation, where freezing tolerance is enhanced by a prior exposure to low non-freezing temperatures (Thomashow, 1999). The *dehydration-responsive element binding factor 1* (*DREB1*)/*C-repeat binding factor* (*CBF*) genes in the ERF transcription factor family play a critical role in this cold acclimation in *Arabidopsis thaliana*. Their transcripts are rapidly induced by chilling stress (Gilmour et al., 1998), and the CBF/DREB1 proteins recognize and bind to the C-repeat/dehydration-responsive (CRT/DRE) cis-elements in the promoters of *cold-responsive* (*COR*) genes to activate *COR* genes expression for freezing tolerance (Stockinger et al., 1997; Liu et al., 1998; Sakuma et al., 2002). Arabidopsis has six *DREB1* genes, namely *DREB1A*/*CBF3*, *DREB1B*/*CBF1*, *DREB1C*/*CBF2*, *DREB1D*/*CBF4*, *DREB1E*/*DDF2*, and *DREB1F*/*DDF1* (Sakuma et al., 2002). Three of them, *CBF1*, *CBF2*, and *CBF3* genes, are shown to be required for freezing tolerance and cold acclimation by the study of knockout mutant combinations generated by genome editing (Hua, 2016). The *cbf1 cbf2 cbf3* triple mutants were more susceptible to freezing than the wild type (WT) with prior low temperature treatment (Jia et al., 2016; Zhao et al., 2016). Without prior treatment, the *cbf1 cbf2 cbf3* mutants did not exhibit more susceptible to freezing (Jia et al., 2016) or was only slightly more susceptible under one out of eight freezing temperatures tested (Zhao et al., 2016). The three Arabidopsis CBF/DREB1 proteins are predicted to positively regulate expression of a total of 346 and 112 *COR* genes in two independent studies (Jia et al., 2016; Zhao et al., 2016). Recently, 146 Arabidopsis genes are defined as direct CBF/DREB1 targets by chromatin immunoprecipitation (ChIP) combined with a transcriptional analysis, and these genes are involved in abiotic stress responses, hormone signaling, and environmental signaling (Song et al., 2021).

*CBF*/*DREB1* genes are also implicated in tolerance to other abiotic stresses. Compared to the WT, the Arabidopsis *cbf1 cbf2 cbf3* triple mutant is more susceptible to salt (Zhao et al., 2016), and *cbf4* mutant is more susceptible to drought (Vonapartis et al., 2022). Overexpression of the Arabidopsis *DREB1A*, *DREB1B*, and *DREB1C* enhanced tolerance to freezing, salt, and drought in Arabidopsis (Liu et al., 1998; Kasuga et al., 1999; Gilmour et al., 2004). Overexpression of the Arabidopsis *DREB1D* enhanced drought tolerance in soybean (Guttikonda et al., 2014), while overexpression of the Arabidopsis *DREB1F*/*DDF1* enhanced tolerance to freezing, drought, and heat in Arabidopsis (Kang et al., 2011). The function of *DREB1* genes in other plants have also been investigated. Overexpression of *DREB1* genes from cotton, soybean, and zoysia grass was shown to enhance tolerances to chilling, drought, heat, and salt (Gao et al., 2005; Shan et al., 2007; Kidokoro et al., 2015; Feng et al., 2019; Zhou et al., 2020). These results suggest that DREB1 proteins could regulate tolerance to multiple abiotic stresses.

The roles of rice *DREB1* genes have been investigated mostly by overexpression studies. Overexpression of four rice DREB1 genes is shown to enhance stress tolerance: *OsDREB1A* for freezing and high salt tolerances in Arabidopsis (Dubouzet et al., 2003), *OsDREB1D* for tolerances to freezing and high-salt in Arabidopsis (Zhang et al., 2009), *OsDREB1F* for tolerances to salt, drought, and cold in both Arabidopsis and rice (Wang et al., 2008), and *OsDREB1G* for chilling tolerance in rice (Moon et al., 2019). The only study of *DREB1* function by its loss of function (LOF) mutant is on *OsDREB1A* which was shown to be a positive regulator of cold-induced calcium influx and chilling tolerance in rice (Wang et al., 2021).

In this study, we investigated the roles of three rice *DREB1* genes (*OsDREB1C*, *E*, and *G*) which are the closest homologs of the three Arabidopsis *DREB1* genes (*CBF1*, *CBF2,* and *CBF3*) by generating and analyzing their LOF mutants. These genes are found to be positive regulators of chilling tolerance but do not affect cold acclimation. They are also found to promote tolerance to other abiotic stresses including heat, salt and drought in rice. Transcriptome analysis suggests that these *OsDREB1* genes affect ROS scavenging and cell death. Thus, our study uncovers function of *OsDREB1C*/*E*/*G* in multiple abiotic stress tolerance in rice and reveals potential similar and distinct functions of these genes in rice and Arabidopsis.

## Materials and Methods

### Phylogenetic and Expression Analyses

Protein sequences of *DREB1* genes from Arabidopsis and rice were aligned using ClustalX. A neighbor-joining tree with a bootstrap setting of 1,000 was generated using MEGA7.0.14 (Kumar et al., 2016). Expression data of *OsDREB1* genes in response to abiotic stresses were obtained from GENEVESTIGATOR.^1^

### Mutant Generation and Identification

All rice plants in this study are *Oryza sativa* cv. Nipponbare (NIP). Mutants were generated in NIP using CRISPR/Cas9 system (Xing et al., 2014). Specific gRNA targets were designed using CRISPR-PLANT website.^2^ The gRNA targets were cloned into the pHUE411 vector (Xing et al., 2014), and the resulting constructs were transformed into NIP by Agrobacterium-mediated transformation (Nishimura et al., 2006). Genomic fragments around the target sites in the mutants were amplified by PCR, and the edited mutations were identified by sequencing the PCR products. The homozygous mutants were selected by PCR based genotyping. All the primers used in this study are listed in Supplementary Table 1.

### Plant Growth Conditions and Abiotic Stress Treatments

All plants were grown in growth chamber with a photoperiod of 16h-light/8h-dark and at 28°C unless specified (such as for stress treatment). Seeds were sterilized with 5% bleach for 10 min followed by three washes with water. The sterilized seeds were soaked in water at 28°C for 3 days for germination. For hydroponic growth, seeds at a similar germination stage were transferred to a 96-well plate with the bottom cut out. Seedlings were grown hydroponically till the third leaf just emerged, and then cultured in Yoshida nutrient solution (Yoshida et al., 1971). For soil growth, the germinated seeds were transferred from water to soil (nutrient soil:vermiculite = 3:1) and grown in growth chamber.

All stress treatments were carried out in growth chambers. For chilling treatment, the hydroponically grown seedlings at three-leaf stage (when the third leaf was fully expanded) were subject to 6°C treatment for 2.5–4 days and then returned to 28°C for 7 days for recovery growth. For suboptimal low temperature treatment, germinated seeds were transferred to soil and grown under 16°C. For cold acclimation treatment, seedlings at three-leaf stage (grown hydroponically at 28°C) were subject to 1 day of 12°C growth (cold acclimation, CA) or kept at 28°C for 1 day (no acclimation, NA). They were then subject to a 6°C treatment for 3.5–4 days before a 7-day recovery growth at 28°C. For heat treatment, the soil grown seedlings at three-leaf stage were treated at 48°C for 2 days and then returned to 28°C for 7-day recovery growth. For salt treatment, the soil grown seedlings of 3-day-old were treated with 200 mM NaCl for 21–23 days. For drought treatment, the soil-grown seedlings at three-leaf stage were withheld of watering for 10 days and then re-watered for 7 days for recovery growth. For survival analysis, 24 hydroponically grown seedlings or 21 soil growth seedlings were used in a biological repeat. At least three biological replicates were performed for each analysis.

### DAB and Trypan Blue Staining

Accumulations of H_2_O_2_ and cell death were detected by 3,3′-diaminobenzidine (DAB) staining and trypan blue staining, respectively, as previously described (Bach-Pages and Preston, 2018). The second leaves of the soil grown seedlings at three-leaf stage were used.

### Transcriptome Analysis

The three-leaf stage seedlings that were soil grown at 28°C were treated at 6°C for 0, 4, and 24 h before all above ground tissue of three biological replicates were collected. Total RNA was extracted from tissues using an RNA extraction kit (RP1002, Bioteke Corporation). RNA sequencing (RNA-seq) was carried out at Berry Genomics.^3^ Approximately 6.0 GB of raw reads were generated from each sample. The clean reads were mapped to rice genome,^4^ and expression level for each gene was determined by its relative abundance or value of fragments per kilobase of exon model per million mapped (FPKM). Gene expression values in FPKM was used for principal component analysis.^5^ Differentially expressed genes (DEGs) were identified using edgeR package (3.32.1) by R Language (R Core Team, 2021). DEGs between two samples were defined by fold change ≥ 2 and false discovery rate (FDR) ≤ 0.01. Heat maps were plotted *via* TBtools or using pheatmap (1.0.12) and ggplot2 (3.3.3) packages by R language (Chen et al., 2020; R Core Team, 2021). Venn diagrams were plotted using Venn Diagram package (1.6.20) by R language (R Core Team, 2021). Gene ontology (GO) enrichment analysis was performed by PANTHER.^6^ GO annotation was downloaded from RiceXPro.^7^ The promoter analysis was performed by a manual search for the DRE/CRT motif (G/ACCGAC) in the 1.5 kb fragment 5′ to the translation start codon of the candidate gene. The RNA-seq data were submitted to National Center for Biotechnology Information (accession number: PRJNA797855).^8^

## Results

### Phylogenetic and Expression Analyses of the *OsDREB1* Genes in Rice

**Figure 1:**
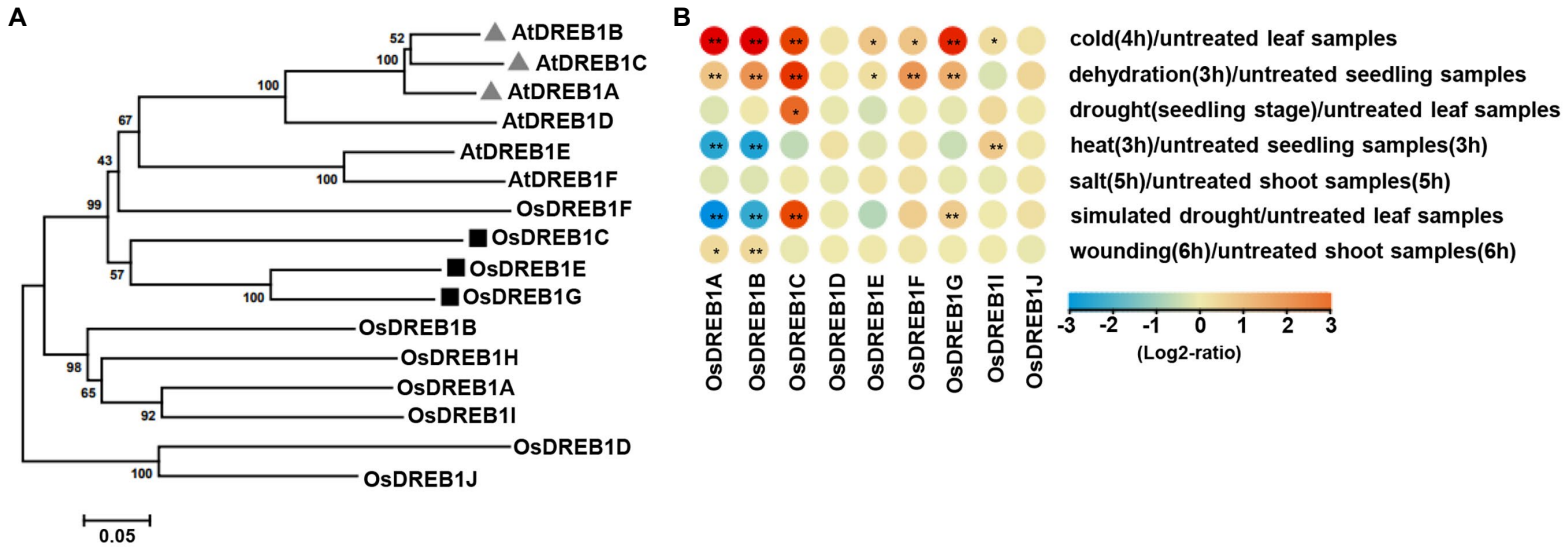
Expression patterns and phylogenetic analyses of *OsDREB1* genes in rice. **(A)** Phylogenetic tree of the OsDREB1 and AtDREB1 proteins. The protein sequences were aligned using ClustatW method, and the neighbor-joining tree with a bootstrap setting of 1,000 was generated using MEGA7.0.14 software. The genes marked with black targets are discussed in this study. The AtDREB1 and OsDREB1 protein sequences were available by TAIR (https://www.arabidopsis.org/) and RiceXPro (https://ricexpro.dna.affrc.go.jp/), respectively. **(B)** Expression patterns of *OsDREB1* genes in response to abiotic stresses. Heat map was plotted *via* Tbtools. Expression levels are given as log2 ratio values. Expression values are obtained from GENEVESTIGATOR (https://genevestigator.com/). * and ** indicate significant differences compared to no treatment at *p* < 0.05 and *p* < 0.01, respectively, by Student’s *t*-test.

The *DREB1* family has six members in Arabidopsis and 10 members (named *OsDREB1A* to *OsDREB1J*) in rice.^9^ Analysis of genes from 38 organisms revealed that this gene family diverged before the separation of Arabidopsis and rice, and the *DREB1* genes have independently amplified in two species (Supplementary Figure 1). This suggests that the three *CBF* genes in Arabidopsis do not have a one-to-one orthologous *DREB1* genes in rice. Protein sequence homology analysis of the six Arabidopsis and 10 rice *DREB1* genes, however, revealed that *OsDREB1C*, *OsDREB1E*, *OsDREB1F*, and *OsDREB1G* are likely closer to the six Arabidopsis *DREB1* genes in sequence identity (Figure 1A).

We further examined the expression patterns of these 10 *OsDREB1* genes (except for *OsDREB1H* which is absent in the database) using the curated high-quality transcriptome data at GENEVESTIGATOR (see Footnote^1^). All these nine *OsDREB1* genes (*1A*, *1B*, *1C*, *1D*, *1E*, *1F*, *1G*, *1I,* and *1J*) have altered expression in response to one or multiple abiotic stresses. Chilling treatment (4°C for 4 h) increased the expression of *1A*, *1B*, *1C*, *1E*, *1F*, *1G* and *1I*. Dehydration treatment (on 3 mm Whatman paper for 3 h) increased the expression of *1A*, *1B*, *1C*, *1E*, *1F,* and *1G*. Drought treatment (no watering till leaves were completely rolled) increased the expression of *1C*. Heat treatment (42°C for 3 h) increased expression of *1I* and repressed the expression *1A* and *1B*. Salt treatment (300 mM NaCl for 5 h) had no significant effect on the expression of any of these nine *OsDREB1* genes. Simulated drought treatment (25% polyethylene glycol6000 for 1 h) increased the expression of *1C* and *1G* and repressed the expression *1A* and *1B*. Wounding treatment (needle puncturing 15 times in 6 h) increased expression of *1A* and *1B* (Figure 1B). Therefore, most of the *OsDREB1* genes had increased expression in response to chilling and dehydration stresses.

### Generation of *dreb1* Mutants in Rice

We selected three *OsDREB1* genes (*OsDREB1C*, *OsDREB1E,* and *OsDREB1G*) for further functional analysis because they are more related to each other and are more closely related to the Arabidopsis *DREB1* genes than six other rice genes (Figure 1A). LOF mutants of these three genes were generated in the NIP background by using CRISPR/Cas9 gene editing system (Xing et al., 2014). Two gRNA targets were used to generate single mutants of *dreb1c* (*via* targets C1, C2), *dreb1e* (*via* targets E1, E3), and *dreb1g* (*via* targets G2, G3). A total of four *dreb1c* mutant alleles (*dreb1c-1*, *dreb1c-2*, *dreb1c-3,* and *dreb1c-4*), two *dreb1e* alleles (*dreb1e-1* and *dreb1e-2*), and one *dreb1g* mutant (*dreb1g-1*) were obtained (Table 1). A gRNA target (C2) specific for *OsDREB1C* and gRNA targets (E2, G1) for both *OsDREB1E* and *OsDREB1G* was used to generate the *dreb1ceg* triple mutant (Table 1).

**Table 1:** Mutations in rice *DREB1c*, *DREB1e*, and *DREB1g* genes generated *via* the CRISPR/Cas9 system.

Gene (mutant)	Nucleic acid mutation		Predicted impact on protein
*OsDREB1C* (*dreb1c-1*)	ACC - - - - - - - - - -// - - - - - - - - -GCC	1 bp insertion after 11 bp and a deletion from 13 to 279 bp (including targets C1 and C2)	Reading frame shift after amino acid (aa) 4
*OsDREB1C* (*dreb1c-2*)	GAGTACGCGACGG - - - CGT TGCCTCAACTTCGCCGAACT	3 bp deletion at target C1; 1 bp increase at target C2	Altered at aa 12-13 and reading frame shift after aa 91
*OsDREB1C* (*dreb1c-3*)	GAGTACGCGACGGTGATCGT TGCCTCAACTTCGCCGAACT	1 bp increase at target C1; 1 bp increase at target C2	Reading frame shift after aa 12 and coding termination after aa 147
*OsDREB1C* (*dreb1c-4*)	GAGTACGCGA - - - - - // - - -GGA TGCCTCAACTTCGCCGAACT	36 bp deletion at target C1; 1 bp increase at target C2	Deletion of aa 11-22 and reading frame shift after aa 91
*OsDREB1E (dreb1e-1)*	GAGTGGGCGTACTACGGTGGCAG CTTCGCCGACTCGCCGTCGC	3 bp increase at target E1; 1 bp increase at target E3	1 aa insertion at aa 8 and reading frame shift after aa 112
*OsDREB1E (dreb1e-2)*	GAGTGGGCGTACTACGGGCAG CTTCGCCGACTCGCC - CGC	1 bp increase at target E1; 1 bp deletion at target E3	Altered sequence from aa 8 to aa 112
*OsDREB1G (dreb1g-1)*	ACGCGGCACCCCGTGTATCA	1 bp increase at target G2	Reading frame shift after aa 54 and coding termination after aa 134
*OsDREB1C* (*dreb1ceg*)	TGCCTCAACTTCGCC-ACT	1 bp deletion at target C2	Reading frame shift after aa 91 and coding terminated after aa 146.
*OsDREB1E* (*dreb1ceg*)	TCGTCGGGGACGCCGTACGC	1 bp increase at target E2	Reading frame shift after aa 16
*OsDREB1G* (*dreb1ceg*)	TCGTCGGGGACGCCGTCCGC	1 bp increase at target G1	Reading frame shift after aa 17 and coding termination after aa 134

*Nucleic acid mutations (colored in red) and the predicted impact on proteins in the *dreb1c*, *dreb1e*, *dreb1g*, and *dreb1ceg* mutants*.

All these mutants were LOF as predicted by the mutation types. The *dreb1c-1* mutant has an insertion of “C” at position 12 bp (relative to the translation initiation site) and a deletion at 13–279 bp, which are predicted to cause a reading frame shift. The *dreb1c-2* mutant has a deletion of 35–37 bp and an insertion of “A” at 275 bp, leading to a reading frame shift. The *dreb1c-3* mutant has an insertion of “T” and “A” at 38 and 275 bp, respectively, leading to coding termination. The *dreb1c-4* mutant has a deletion at 32–67 bp and an insertion of “A” at 275 bp leading to a reading frame shift. The *dreb1e-1* mutant has an insertion of “TGG” and “T” at 21 and 337 bp, respectively, leading to a reading frame shift. The *dreb1e-2* mutant has an insertion of “G” at 21 bp and a deletion of “G” at 336 bp, leading to protein sequence changes starting from amino acid (aa) positions 8 and 112, respectively. The *dreb1g-1* mutant has an insertion of “A” at 164 bp, leading to coding termination. The *dreb1ceg* mutant has a deletion of “G” at 274 bp in *OsDREB1C*, and an insertion of “A” and “C” at 50 bp in *OsDREB1E* and *OsDREB1G*, respectively, leading to a reading frame shift of *OsDREB1E* and coding terminations of *OsDREB1C* and *OsDREB1G* (Table 1). Homozygous single or triple mutants of these three genes were used for further analyses.

### *OsDREB1C*/*E*/*G* Genes Promote Basal Chilling Tolerance in Rice at Seedling Stage

**Figure 2:**
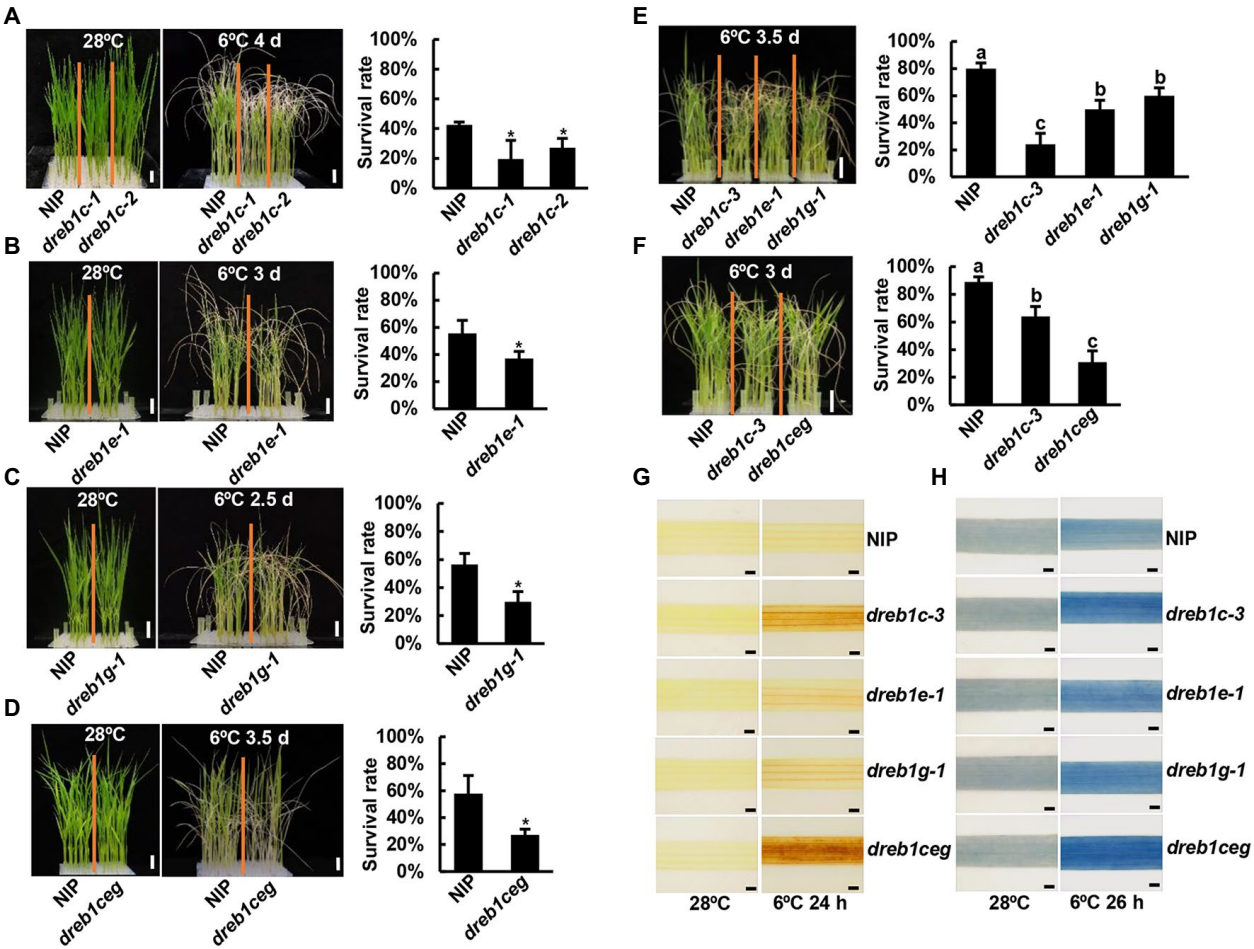
The *dreb1* mutants are more susceptible to chilling at seedling stage. **(A–F)** Growth phenotypes and survival rates of *dreb1c* and WT **(A)**, *dreb1e* and WT **(B)**, *dreb1g* and WT **(C)**, *dreb1ceg* and WT **(D)**, *dreb1c*, *dreb1e*, *dreb1g,* and WT **(E)** as well as *dreb1c*, *dreb1ceg*, and WT **(F)** after chilling treatment and recovery. Seedlings were hydroponically grown at 28°C for 3 weeks, incubated at 6°C (for 2.5–4 days as indicated) and then transferred to normal conditions for 7 days for recovery. Left panels show plant morphology and bars = 2 cm. Right panels show mean values of survival rates from three biological replicates ±SD (standard deviation). Each biological replicate has more than 24 seedlings. Asterisks indicate significant differences compared to NIP (*p < 0.05, Student’s *t*-test). Lowercase letters above the bars indicate significant differences among samples at *p* < 0.05, by SSR-Test. **(G,H)** DAB **(G)** and trypan blue **(H)** staining of the second leaves of *dreb1c-3*, *dreb1e-1*, *dreb1g-1*, *dreb1ceg*, and NIP plants before and after chilling treatment at 6°C for 24 h (DAB) or 26 h (trypan blue). Bars = 1 mm.

The role of *OsDREB1C*/*E*/*G* genes in chilling tolerance was assessed by chilling treatment of the single and triple mutants of *dreb1c*, *dreb1e*, and *dreb1g*. Three-leaf-stage seedlings were subjected to 2.5–4 days of chilling treatment at 6°C and allowed to recover for 7 days. All *dreb1* single mutants had significantly lower survival rates than NIP, although the survival rate of the WT differed among different sets of experiments likely due to a slight variation of temperature and duration of chilling treatment (Figures 2A–F; Supplementary Figure 2). The *dreb1c-1* and *dreb1c-2* mutants had survival rates of 19% and 27%, respectively, lower than the rate of 42% for NIP (Figure 2A). The *dreb1c-3* and *dreb1c-4* mutants had survival rates of 55% and 49%, respectively, lower than the rate of 92% for NIP (Supplementary Figures 2A–C). The *dreb1e-1* mutant had a survival rate of 36%, lower than the rate of 55% for NIP (Figure 2B). The *dreb1e-2* mutant had survival rates of 53%, lower than the rate of 72% for NIP (Supplementary Figures 2D,E). The *dreb1g-1* mutant had a survival rate of 29%, lower than the rate of 56% for NIP (Figure 2C). The *dreb1ceg* triple mutant also had lower survival rate than the wild type NIP (27% versus 57%; Figure 2D). Chilling tolerance levels among *dreb1* single mutants and the triple mutants were also compared. The survival rates of *dreb1e-1* and *dreb1g-1* were 50% and 59%, respectively, higher than the rate of 23% for *dreb1c-3* (Figure 2E). The survival rate of *dreb1c-3* was 63%, higher than the rate of 30% for *dreb1ceg* (Figure 2F). These results indicate that all three rice genes, *OsDREB1C*/*E*/*G*, positively regulate chilling tolerance in rice at seedling stage.

We further analyzed H_2_O_2_ accumulation by DAB staining as ROS accumulation is highly related to chilling tolerance. All *dreb1* mutants had more H_2_O_2_ accumulation in leaves than NIP after 6°C treatment for 24 h. The *dreb1c-3* mutant had more H_2_O_2_ accumulation than *dreb1e-1* and *dreb1g-1*, and *dreb1ceg* had the highest H_2_O_2_ accumulation than all single mutants (Figure 2G). Cell death caused by chilling was subsequently analyzed by trypan blue staining. All *dreb1* mutants had more cell death than NIP after chilling treatment. The *dreb1c-3* mutant had more cell death than *dreb1e-1* and *dreb1g-1*, and *dreb1ceg* had the highest cell death than all single mutants (Figure 2H). Therefore, H_2_O_2_ accumulation and cell death are each associated with chilling susceptibility in the rice *dreb1* mutants.

### *OsDREB1C*/*E*/*G* Genes Promote Rice Growth Under Suboptimal Low Temperature

**Figure 3:**
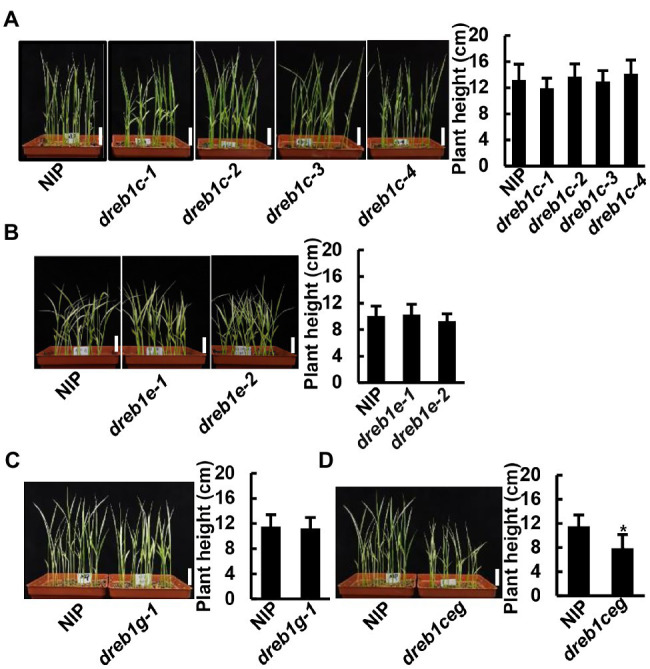
The *dreb1ceg* mutant had reduced growth at seedling stage under suboptimal low temperature. Growth phenotypes and quantification of plant heights of *dreb1c* **(A)**, *dreb1e* **(B)**, *dreb1g* **(C)** and *dreb1ceg* **(D)** grown at 16°C for 32 days. Seeds were germinated at 28°C for 3 days, and then transferred to 16°C for 32 days. Shown is the mean value ±SD of 24 seedlings. Similar results were obtained from two additional biological repeats. Each replicate has 24 seedlings. Asterisks indicate significant differences compared to NIP (*p < 0.05, Student’s *t*-test). Bars in plant images represent 2 cm.

We analyzed the growth phenotypes of the *dreb1* mutants at non-chilling temperatures. Under normal growth temperature (28°C), no growth difference was observed between the *dreb1* mutants and NIP at seedling, tillering, or mature stages (Supplementary Figure 3). Under a suboptimal low temperature (16°C), none of the single *dreb1* mutants showed detectable growth difference compared with NIP at the seedling stage (Figures 3A–C). In contrast, the *dreb1ceg* triple mutant was significantly shorter than the WT NIP at 16°C, with a height of 7.9 cm compared to 11.5 cm of the WT (Figure 3D). These results indicate that the *OsDREB1C*/*E*/*G* genes have an overlapping function in promoting rice growth at suboptimal low temperature.

### *OsDREB1C*/*E*/*G* Genes Do Not Participate in Cold Acclimation in Rice

**Figure 4:**
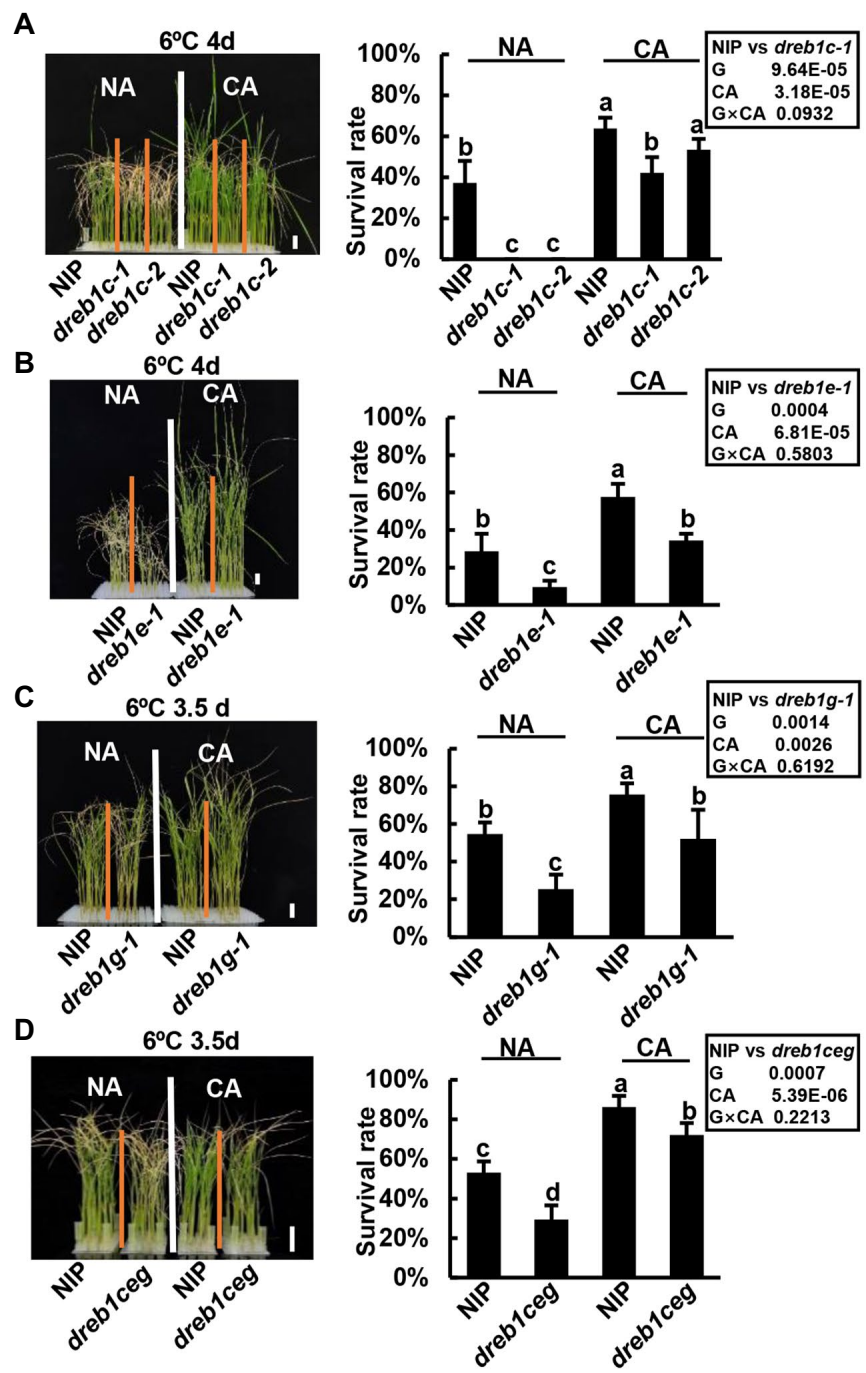
*OsDREB1C*/*E*/*G* genes do not affect cold acclimation in rice. Growth phenotypes and survival rates of *dreb1c* **(A)**, *dreb1e* **(B)**, *dreb1g* **(C)**, and *dreb1ceg* **(D)** after chilling treatment and recovery growth with cold acclimation (CA) and without cold acclimation (NA). Seedlings were hydroponically grown at 28°C for 3 weeks till three-leaf stage, treated 1 day at 12°C (CA) or 1 day at 28°C (NA), before treated at 6°C for 3.5 or 4 days followed by a recovery growth at 28°C for 7 days. Left panels show plant morphology after recovery. Bars in plant images represent 2 cm. Right panels show survival rates expressed as mean ± SD from three biological replicates each with 24 seedlings. Lowercase letters above the bars indicate significant differences among samples at *p* < 0.05, by SSR test. Insert shows multiway-ANOVA analysis of genotype (NIP versus *dreb1* mutants) and cold acclimation (NA versus CA).

We further determined whether *OsDREB1C*/*E*/*G* genes regulate cold acclimation. The WT NIP plants exhibited a higher survival rate at 6°C with a prior 12°C treatment of 1 day compared to no prior treatment (Figure 4), indicating that rice has cold acclimation for chilling tolerance. All the *dreb1* mutants also exhibited cold acclimation. In one set of experiment, NIP, *dreb1c-1,* and *dreb1c-2* had survival rates of 63%, 42%, and 53%, respectively, with prior cold treatment (cold acclimation, CA), higher than the rates of 37%, 0%, and 0% observed with no prior cold treatment (no acclimation, NA; Figure 4A). NIP and *dreb1e-1* had survival rates of 57% and 34%, respectively, with CA, higher than the rates of 28% and 9% with NA (Figure 4B). NIP and *dreb1g-1* had survival rates of 75% and 52%, respectively, with CA, higher than the rates of 54% and 25% with NA (Figure 4C). NIP and *dreb1ceg* had survival rates of 86% and 72%, respectively, with CA, higher than the rates of 53% and 29% with NA (Figure 4D). With CA, all mutants including *dreb1c-1*, *dreb1e-1*, *dreb1g-1*, and *dreb1ceg* plants showed more chilling susceptibility compared with WT NIP (Figures 4A–D). The only exception was *dreb1c-2* which had a lower survival rate compared with NIP, but the difference was not significant (Figure 4A). This indicates that *OsDREB1C/E/G* genes do not affect cold acclimation in rice. We further analyzed the genotype by environment (G x E) interaction between each genotype pair (WT versus the *dreb1* mutants) and cold acclimation (NA versus CA) on survival rates. No significant interaction was found for any of the *OsDREB1* genotype and CA, indicating that the *dreb1* mutants have a cold acclimation-independent chilling susceptibility (Figure 4). These results indicate that *OsDREB1C*/*E*/*G* are not important for cold acclimation in rice, or they have overlapping functions with other *OsDREB1* genes in cold acclimation.

### Transcriptome Analysis of the *dreb1* Mutants

To further understand how *OsDREB1* regulate chilling tolerance in rice, we carried out RNA-seq analysis on the WT, *dreb1c-2*, *dreb1g-1,* and *dreb1ceg* plants at 3-leaf-stage before and after a chilling treatment of 6°C. Principal component analysis (PCA) revealed that the WT and the *dreb1* mutants were similar to each other at any time points in the first two principal components, and chilling treatment was the most important factor in differentiating the samples (Supplementary Figure 4).

This PCA showed that chilling treatment induced a similar global transcriptional change in WT and *dreb1* mutants. A total of 747 chilling up-regulated and 406 chilling down-regulated DEGs were shared among the WT, *dreb1c-2*, *dreb1g-1*, and *dreb1ceg* at 4 h of 6°C treatment, accounting for 69%, 56%, 56%, and 70% of up-regulated DEGs as well as 52%, 45%, 42%, and 52% of down-regulated DEGs in the WT, *dreb1c*, *dreb1g*, and *dreb1ceg*, respectively (Supplementary Figure 5). Moreover, 2,774 up-regulated and 2,148 down-regulated DEGs were shared among the WT and three *dreb1* mutants at 24 h, accounting for 77%, 84%, 71%, and 71% of up-regulated DEGs as well as 64%, 65%, 59%, and 57% of down-regulated DEGs in the WT, *dreb1c*, *dreb1g*, and *dreb1ceg*, respectively (Supplementary Figure 5). These genes represent the majority of the DEGs in WT or *dreb1* mutants, indicating that the *dreb1* mutations do not drastically alter chilling-induced or chilling-repressed DEGs. At 4 h, 895 up-regulated and 565 down-regulated DEGs were identified in both *dreb1g* and *dreb1ceg*, and 874 up-regulated and 506 down-regulated DEGs were identified in both *dreb1c* and *dreb1ceg* (Supplementary Figure 5). At 24 h, 3,419 up-regulated and 2,960 down-regulated DEGs were identified in both *dreb1g* and *dreb1ceg*, and 2,962 up-regulated and 2,570 down-regulated DEGs were identified in both *dreb1c* and *dreb1ceg* (Supplementary Figure 5). The *dreb1g* mutant shared more DEGs with *dreb1ceg* than *dreb1c*, suggesting that *OsDREB1G* has a larger functional contribution than *OsDREB1C* to gene expression changes under chilling.

Considering that *OsDREB1* genes are likely transcriptional activators, we examined genes that had a reduced expression in the *dreb1ceg* mutant under chilling because they are potentially regulatory target genes of *OsDREB1C*/*E*/*G*. These include chilling up-regulated DEGs in WT but not in the triple mutant as well as chilling down-regulated DEGs in *dreb1ceg* but not in WT at 4 h and/or 24 h. These DEGs were analyzed for enrichment of Gene Ontology (GO) terms defined by FDR < 0.05. No enriched GO terms were identified for the 428 up-regulated DEGs that appeared in WT but not in *dreb1ceg* (Supplementary Figure 6A). The 1,092 down-regulated DEGs that appeared in *dreb1ceg* but not in WT were enriched for the GO terms “plant-type hypersensitive response”, “protein maturation”, and “peptidyl-amino acid modification”, with “plant-type hypersensitive response” having the highest fold enrichment of 24 (Supplementary Figures 6B,C). Under the GO term “plant-type hypersensitive response” were four genes that had a lower expression at 24 h in *dreb1ceg* compared to the WT (Supplementary Figure 6D). These four genes code for a transporter, a ubiquitin-protein ligase, and two MACPF domain-containing protein. One MACPF domain containing protein CAD1 is a negative regulator of the cell death in Arabidopsis, and its mutant exhibited a hypersensitive response-like cell death (Morita-Yamamuro et al., 2005). The ubiquitin-protein ligase *OsNLA1* modulates phosphate accumulation in rice, and the loss of its function leads to lethality (Zhong et al., 2017). This suggests that the LOF of *OsDREB1C*/*E*/*G* leads to reduced expression of potential negative regulators of cell death, which might contribute to the increased cell death in the *dreb1ceg* mutant under chilling.

Chilling downregulated 3,568, 3,534, 4,006, and 4,006 genes in WT, *dreb1c*, *dreb1g,* and *dreb1ceg*, respectively (Supplementary Figure 7A). GO analysis on downregulated DEGs in WT and the three *dreb1* mutants was performed to identify differential GO terms between WT and the mutants. One GO term “hydrogen peroxide catabolic process” was present in all *dreb1* mutants but not in WT (Supplementary Figure 7B). Under this enriched GO term were 18 chilling downregulated DEGs (1 peroxiredoxin-2E-1, 17 peroxidase) which had a lower expression in both WT and triple mutant at 24 h compared to 0 h. Under this GO term were also 10 chilling downregulated genes (one peroxiredoxin-2E-1, nine peroxidase) that had a lower expression in *dreb1ceg* compared to the WT at 24 h (Supplementary Figure 7C). Therefore, more genes that are involved in ROS scavenging have a lower expression in the *dreb1* mutant compared to the wild type at 24 h. Plant peroxidase scavenges ROS and protects host cells from damages made by excessive ROS (Liu et al., 2021a). These results suggest that *OsDREB1C*/*E*/*G* may promote ROS scavenging under chilling.

### Prediction of Potential Target Genes of OsDREB1C/E/G

As transcriptional factors, OsDREB1 proteins are expected to bind to specific cis-element motifs in the promoter region of their target genes and affect their gene expression. Because AtDREB1s mainly function as transcriptional activators (Song et al., 2021), we searched among genes with reduced expression in the *dreb1ceg* mutant compared to the WT for potential direct regulatory targets of OsDREB1C/E/G by the criterion of having DRE/CRT element(s) in their promoter region. We first examined the genes under GO terms uniquely enriched in the mutant but not the WT for chilling down regulated DEGs. Among the four genes under the GO term “plant-type hypersensitive response”, one gene (LOC_Os07g07194, MACPF domain-containing protein) contained a DRE/CRT (G/ACCGAC) element in its promoter region. Among the 18 genes under the term “hydrogen peroxide catabolic process”, three genes (LOC_Os03g22020, LOC_Os06g42000, and LOC_Os11g43980) contained one DRE/CRT element in their promoter regions. Therefore, these four genes are potential direct targets of OsDREB1C/E/G.

**Figure 5:**
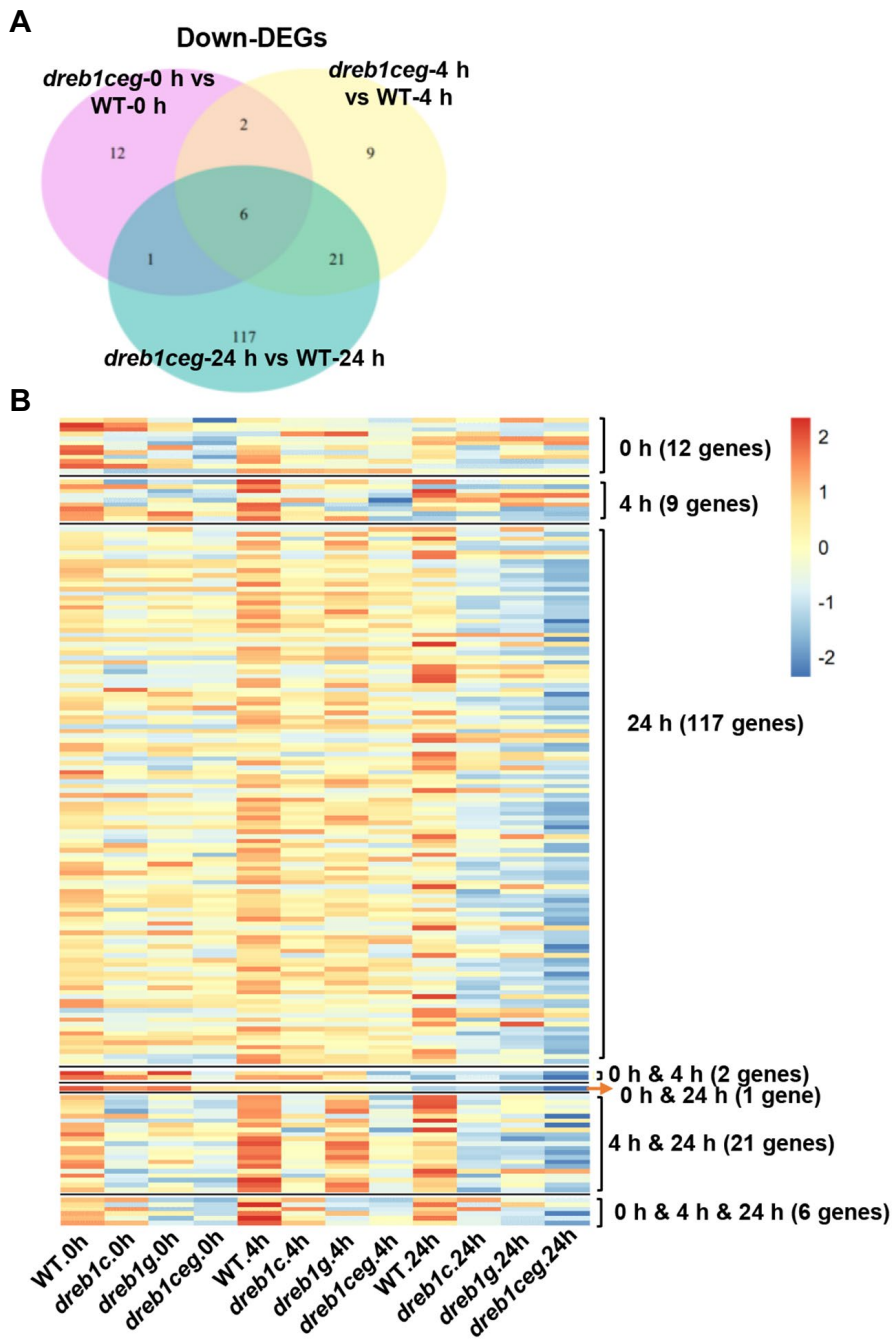
Transcriptome analysis of the *dreb1* mutants. **(A)** Venn diagram indicates the number of downregulated DEGs (fold change ≥ 2, FDR ≤ 0.01) of *dreb1ceg* versus the WT at 0, 4, and 24 h after chilling treatment at 6°C. **(B)** Heat map of expression levels of 168 OsDREB1C/E/G-activated genes in the WT and the *dreb1* mutants at 0, 4, and 24 h after chilling treatment. The OsDREB1C/E/G-activated genes are defined by a reduced expression in *dreb1ceg* compared to the WT (fold reduction ≥ 2, FDR ≤ 0.01) at any of the three time points. Heat map is plotted using pheatmap package (1.0.12) and ggplot2 (3.3.3) by R language. Venn diagram is plotted using VennDiagram package (1.6.20) by R language.

In addition, we examined genes with reduced expression in *dreb1ceg* compared to the WT at each of the three time points (0, 4, and 24 h) of chilling treatment. With 21, 38, and 145 downregulated DEGs (mutant versus WT) at 0, 4, and 24 h, respectively, the *dreb1ceg* mutant had a total of 168 downregulated DEGs compared to the WT with three time points combined (Figure 5A). These genes were defined as potential OsDREB1C/E/G target genes.

The potential function of these 168 potential OsDREB1C/E/G target genes was subject to GO analysis. Among the 95 genes that had a GO annotation in the biological process, no enrichment of GO terms was identified for these genes. However, many of these genes have shared GO terms. Seventy genes had the GO term “metabolic process”, including carbohydrate metabolism, protein metabolism, lipid metabolism, photosynthesis, organic acid metabolism, protein modification process and nucleobase, nucleoside, nucleotide, and nucleic acid metabolic process (Supplementary Figure 8). A total of 33, 16, 37, 7, and 14 genes had the GO terms of “response to stimulus”, “transport”, “cellular process”, “signal transduction”, and “developmental process”, respectively (Supplementary Figure 8). Sixteen and 26 genes had the GO terms of “response to abiotic stimulus”, and “response to stress”, respectively. Two genes were under the term “cell death,” which is a related term of “plant-type hypersensitive response”.

We further examined the expression pattern of these 168 potential OsDREB1C/E/G target genes to determine whether their regulation by *OsDREB1C*/*E*/*G* genes is time-point specific. Heat map analysis revealed that these genes, although identified from a specific time point, mostly had a reduced expression at all three time points (Figure 5B). For the 12 DEGs of *dreb1ceg* that are unique at 0 h, almost all also had a lower expression in *dreb1c* (11 genes) and *dreb1g* (12 genes) than in WT. Ten of these genes had a lower expression in the *dreb1c*, *dreb1g*, and *dreb1ceg* mutants compared to WT at 4 h, and most of them (nine for *dreb1c*, seven for *dreb1g,* and five for *dreb1ceg*) had a lower expression at 24 h. A similar low specificity for time points was also observed for DEGs at 4 and 24 h where they had lower expression at the other two time points in the mutants compared to the WT. In sum, the majority of these genes had decreased expression in the *dreb1ceg* mutants at all three time points although the fold change is less than 2 and were therefore were not identified as DEGs at all time points.

Next, we search for the presence of sequence motif G/ACCGAC, the DRE/CRT element, in the promoter region of the potential OsDREB1C/E/G target genes. Because some genes do not have defined transcription initiation sites, we used the 1.5 kb fragment 5′ to the translation start codon of these genes as a proxy for promoter fragment. Among the 168 genes, 53 genes contained at least one DRE/CRT element in their promoter region, with 15 genes containing more than one element (Supplementary Table 2). Eight genes have only one element within the 100 bp region 5′ to the translation start codon (Supplementary Table 2), and whether those are in the 5’ UTR region needs further investigation. Function of ten out of these 53 genes have been previously studied. Four of them, *OsHAN1*, *OsDREB1F*, *OsNAP,* and *OsGNA1*, have been shown to be involved in chilling tolerance or growth. The *OsHAN1* gene codes for an oxidase, and it LOF mutant had enhanced chilling tolerance (Mao et al., 2019). *OsDREB1F* is member of DREB1 family, and its overexpression has been shown to enhance chilling tolerance in rice (Wang et al., 2008). It is interesting that *OsDREB1F* is a potential target of the OsDREB1C/E/G proteins in the same family. *OsNAP* encodes a NAC transcription factor, and its overexpression also enhances chilling tolerance rice (Chen et al., 2014). *OsGNA1*, encoding a glucosamine-6-phosphate acetyltransferase, and its LOF mutant has a temperature-sensitive defect in root elongation (Jiang et al., 2005). In addition, *OsGRX2* encodes glutaredoxin that is involved in detoxification processes (Jeong et al., 2018) and could contribute to stress tolerance. Five other genes are involved in growth and developmental processes. *OsDof18* is involved in growth and ammonium transport regulation (Wu et al., 2017), *OsDjA7/8* is essential for chloroplast development (Zhu et al., 2015), *OsNIA2*, encoding a nitrate reductase, increases nitrogen uptake capacity (Sun et al., 2015), *OsMYB103L* is involved in GA-mediated regulation of secondary wall biosynthesis (Ye et al., 2015), and *OsSPW1* controls floral organ identity (Nagasawa et al., 2003). Thus, these potential target genes may contribute to chilling tolerance and chilling growth mediated by OsDREB1C/E/G.

### *OsDREB1C*/*E*/*G* Genes Promote Tolerance to Heat, Salt and Drought at Seedling Stage

To determine whether these *OsDREB1C*/*E*/*G* genes also play a role in tolerance to other abiotic stresses in rice, the *dreb1* mutants were subjected to heat, salt, and drought treatments. The survival rates for the WT NIP and the mutant *dreb1* plants grown in the same pot were compared in the same pot to control variability of stress treatment.

**Figure 6:**
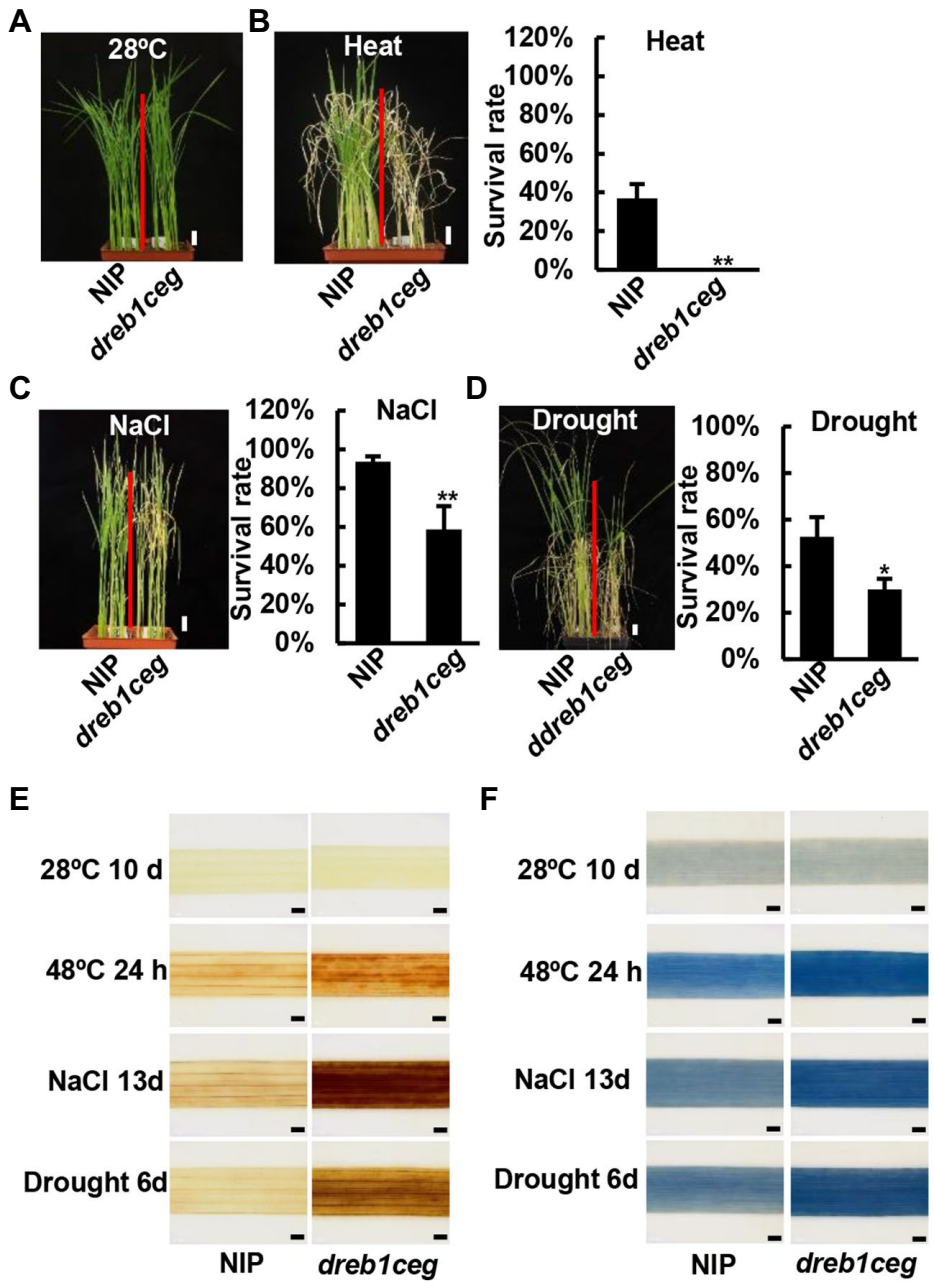
The *dreb1ceg* mutant is more susceptible to heat, salt and drought stress at seedling stage. **(A)** Growth phenotype of NIP and *dreb1ceg* under control condition of 28°C. **(B)** Growth phenotype and survival rates of NIP and *dreb1ceg* after heat treatment (48°C for 2 days) and 7-day recovery growth at 28°C. **(C)** Growth phenotype and survival rates of NIP and *dreb1ceg* after salt treatment (200 mM NaCl) for 23 days. **(D)** Growth phenotype and survival rates of NIP and *dreb1ceg* after drought treatment for 10 days followed by a 7-day recovery. **(E,F)** DAB **(E)** and trypan blue **(F)** staining of the second leaves of NIP and *dreb1ceg* under control condition (28°C), heat (48°C for 24 h), salt (200 mM for 13 days), and drought stress (6 days of water withheld). Bars in plant images represent 2 cm, and bars in staining images represent 1 mm. Survival rates in **(B–D)** are expressed as mean ± SD from three biological replicates. * and ** indicate significant differences at *p* < 0.05, and *p* < 0.01, respectively, by Student’s *t*-test.

For heat stress, 10-day-old seedlings grown on soil were treated at 48°C for 2 days, followed by a recovery growth. The NIP had a survival rate of 36%, while the *dreb1ceg* mutant plants all died (Figures 6A,B). These data indicate that *OsDREB1C*/*E*/*G* genes are positive regulators of tolerance to heat in rice.

For salt stress, 3-day-old seedlings were treated with 200 mM NaCl for 21 or 23 days. The survival rates for the four *dreb1c* mutants were 76%, 71%, 57%, and 61%, respectively, lower than the rate of 92% for NIP (Supplementary Figure 9A). No significant difference in salt tolerance was observed between *dreb1e* and NIP (Supplementary Figure 9B). The survival rate for *dreb1g-1* was 74%, lower than the rate of 96% for NIP (Supplementary Figure 9C). The survival rate for *dreb1ceg* was 58%, lower than the rate of 93% for NIP (Figure 6C). We further compared salt tolerance of the single and triple mutants. The survival rates for *dreb1c-1*, *dreb1g-1*, and *dreb1ceg* were 42%, 41%, and 35%, respectively, all lower than the rate of 64% for NIP (Supplementary Figure 9D). However, no significant difference was observed between single and triple mutants (Supplementary Figure 9D). These results indicate that the *OsDREB1C* and *OsDREB1G* genes, but not the *OsDREB1E* gene, are positive regulators of salt tolerance in rice.

For drought stress, seedlings at three-leaf stage were withheld of water for 10 days and then re-watered for 7 days. No significant difference in survival rate was observed among *dreb1c-2*, *dreb1e-1,* and NIP (Supplementary Figures 10A,B). The survival rate for the *dreb1g-1* mutant was 17%, lower than 53% for NIP (Supplementary Figure 10C). The survival rate for the *dreb1ceg* mutant was 29%, lower than 52% for NIP (Figure 6D). Compared drought tolerance between the *dreb1g* and *dreb1ceg* mutant, no significant difference was observed (Supplementary Figure 10D). These data indicate that the *OsDREB1G* gene but not *OsDREB1C* or *OsDREB1E* positively regulates drought tolerance in rice.

Accumulation of ROS and cell death was examined in the *dreb1ceg* mutant after each of the stress treatments: 24 h of 48°C, 13 days of 200 mM NaCl, and 6 days of water withheld. DAB staining revealed a higher H_2_O_2_ accumulation in leaves of the *dreb1ceg* mutant compared to that in NIP after all three stress treatments: heat, salt, and drought (Figure 6E). Similarly, leaves of the *dreb1ceg* mutant had more cell death than NIP after these stress treatments as indicated by trypan blue staining (Figure 6F). Therefore, *OsDREB1C*/*E*/*G* genes function to repress ROS overaccumulation and cell death under heat, salt, and drought stresses.

## Discussion

DREB1 transcription factors are implicated in stress tolerance, especially cold tolerance. Three members in Arabidopsis, *AtDREB1A*, *AtDREB1B,* and *AtDREB1C*, are key regulators of cold acclimation for freezing tolerance, while *AtDREB1D* is a positive regulator of drought tolerance. The function of DREB1 proteins in other plant species are largely unexplored except for some overexpression studies in heterologous systems. Here, we report the function of three of the 10 *DREB1* genes in rice, *OsDREB1C*/*E*/*G*, through mutant characterizations. These three genes are found to be critical for basal chilling tolerance and low temperature growth in rice. They are also important for stress tolerance to heat, salt and drought. These indicate that *DREB1* genes have conserved function in stress tolerance in higher plants. These findings offer an explanation on previous observations that stress tolerance can be improved by overexpressing *DREB1* genes in heterologous systems.

The rice *OsDREB1C*/*E*/*G* genes, similar to the Arabidopsis *CBF1/2/3* genes, are positive regulators of cold tolerance. However, the Arabidopsis *AtDREB1A*/*B*/*C* genes are required for cold acclimation but not significantly for basal freezing tolerance (Jia et al., 2016). In contrast, the rice *OsDREB1C*/*E*/*G* genes are required for basal chilling tolerance but not for cold acclimation (Figure 4). This may suggest a species-specific function of *DREB1* genes in basal cold tolerance and acclimation-induced cold tolerance. It could also result from an expansion of the *DREB1* gene family in rice, and other *OsDREB1* genes may function in cold acclimation or have overlapping function with *OsDREB1C*/*E*/*G* in cold acclimation. Further investigation on other *OsDREB1* genes, especially *OsDREB1F*, could resolve this question. Alternatively, this differential function in cold acclimation may reflect a difference of cold tolerance between Arabidopsis and rice. Arabidopsis is chilling tolerant (surviving at 4°C), while rice is chilling susceptible (not surviving at 4°C). Cold acclimation for Arabidopsis happens at chilling temperatures such as 4°C to 10°C, while basal chilling tolerance in rice happens at chilling temperatures in the same range. Therefore, the *DREB1* genes in Arabidopsis and rice genes may function under the same low temperature range.

Strikingly, we found that *OsDREB1C*/*E*/*G* genes are required for stress tolerance to chilling, salt, drought, and heat stress in rice. Although salt and drought tolerances have been reported for some *OsDREB1* genes from overexpression studies, this is the first study reporting a regulation of heat tolerance by any *OsDREB1* genes in rice. Not all three genes have a detectable function in all stress responses. While all three genes promote chilling tolerance, *OsDREB1C* and *OsDREB1G* promote salt tolerance, while *OsDREB1G* promotes drought tolerance. More detailed analysis is needed to reveal if these three genes have overlapping functions in each of the stress tolerance. It will also be interesting to determine the function of other seven *OsDREB1* genes in stress tolerance.

Transcriptome analysis of the rice *dreb1* mutants suggests that chilling tolerance promoted by the *OsDREB1C*/*E*/*G* genes is at least partially through scavenging ROS and reducing cell death. Genes with reduced expression in the *dreb1ceg* mutant compared to the WT were enriched in the GO terms “plant-type hypersensitive response” and “hydrogen peroxide catabolic process” (Supplementary Figures 5, 6). The *dreb1* single mutants, and more so the triple mutant, have more H_2_O_2_ accumulation and cell death than the WT (Figures 2G,H). In addition, the *dreb1ceg* mutant has more H_2_O_2_ accumulation and cell death than the WT after treatment of other abiotic stresses (Figures 6E,F). Together, these results indicate that the *OsDREB1C*/*E*/*G* genes may regulate various abiotic stress responses through shared mechanisms such as ROS scavenging and cell death control. Early studies found a higher ROS accumulation and a more robust ROS response in the more chilling tolerant Japonica rice varieties compared to the less tolerant Indica rice varieties (Zhang et al., 2016). Here, a higher ROS in the *dreb1* mutants compared to the WT (all in Japonica NIP background) is associated with more chilling susceptibility, suggesting that a controlled ROS accumulation is critical for chilling tolerance. This supports the dual role of ROS as signaling molecules and damaging molecules.

This study identified potential target genes that could mediate the function of OsDREB1C/E/G in chilling tolerance or general stress tolerance. A total of 53 potential OsDREB1C/E/G target genes based on the features that they contain the DRE/CRT element in the promoter region and that they have a lower expression in the *dreb1ceg* mutant compared to the WT at 0, 4, or 24 h of chilling treatment. This is likely a stringent criterion because some of the direct target genes of Arabidopsis DREB1 proteins do not have a DRE/CRT element in their promoters (Song et al., 2021). Therefore, more genes could be direct regulatory targets of OsDREB1C/E/G proteins. Nevertheless, some of the 53 candidate target genes could mediate the function of *OsDREB1C*/*E*/*G* in chilling tolerance. Among the 10 genes with functional information, some have been shown to promote chilling tolerance or growth. These genes include *OsDREB1F*, *OsNAP,* and *OsGNA1*, all of which have been shown to be positive regulators of chilling tolerance in rice (Jiang et al., 2005; Wang et al., 2008; Chen et al., 2014). Their lower expression in the *dreb1ceg* mutant after chilling compared to the WT may lead to reduced chilling tolerance. The nitrate reductase coding gene *OsNIA2* could also mediate the function of *OsDREB1C*/*E*/*G* in chilling tolerance because nitrate reductase-regulated NO level is positively associated with freezing tolerance in Arabidopsis (Zhao et al., 2009). *OsHAN1* is a negative regulator of chilling tolerance (Mao et al., 2019). Its lower expression at 0 h but higher expression in *dreb1ceg* at 24 h may contribute to chilling susceptibility. In addition, OsDREB1C/E/G proteins could regulate expression of genes involved in ROS and cell death control. One potential target gene *OsGRX2* encodes glutaredoxin, and its lower expression in *dreb1ceg* could be one of the causes of high accumulation of ROS and cell death associated with more chilling susceptibility. Four other candidate target genes had GO terms of “plant-type hypersensitive response” and “hydrogen peroxide catabolic process,” suggesting that regulation on ROS and cell death could be one major mechanism for DREB1C/E/G to promote chilling tolerance. As regulation of ROS and cell death is also key to tolerance to other abiotic stresses, this could also be one mechanism for these proteins to promote tolerance to heat, drought and salt.

## Conclusion

We revealed an important role of three out of 10 rice *DREB1* genes (*OsDREB1C*/*E*/*G*) in stress tolerance to chilling, heat, salt, and drought in rice. In contrast to the Arabidopsis *CBF1*/*2*/*3* genes, these rice genes are critical for basal chilling tolerance but not cold acclimation. This study also suggests that ROS scavenging and cell death control might be shared mechanisms for these *DREB1* genes to promote tolerance to multiple stresses. This study enhances mechanistic understanding of general stress tolerance in plants and provides genetic materials for enhancing abiotic stress tolerance in crop plants.

## Data Availability Statement

The datasets presented in this study can be found in online repositories. The names of the repository/repositories and accession number(s) can be found at: https://www.ncbi.nlm.nih.gov; PRJNA797855.

## Author Contributions

JH, BZ, HW, and SL designed the experiments and wrote the manuscript. HW and XG performed the experimental work. HW, YJ, and BW processed and analyzed RNA-Seq data. All authors contributed to the article and approved the submitted version.

## Funding

This work is supported by the National Natural Science Foundation of China (31700223 and 31971827), Open Funds of the State Key Laboratory of Plant Physiology and Biochemistry (SKLPPBKF2102), and Jiangsu Collaborative Innovation Center for Modern Crop Production and Cyrus Tang Innovation Center for Crop Seed Industry.

## Conflict of Interest

The authors declare that the research was conducted in the absence of any commercial or financial relationships that could be construed as a potential conflict of interest.

## Publisher’s Note

All claims expressed in this article are solely those of the authors and do not necessarily represent those of their affiliated organizations, or those of the publisher, the editors and the reviewers. Any product that may be evaluated in this article, or claim that may be made by its manufacturer, is not guaranteed or endorsed by the publisher.

## Footnotes

^1^ https://www.genevestigator.com/

^2^ http://www.genome.arizona.edu/crispr/CRISPRsearch.html

^3^ http://www.berrygenomics.com/

^4^ http://plantgdb.org/OsGDB/

^5^ https://www.omicshare.com

^6^ http://www.pantherdb.org/help/PANTHERhelp.jsp

^7^ https://ricexpro.dna.affrc.go.jp/

^8^ https://www.ncbi.nlm.nih.gov/

^9^ http://www.phylogenes.org/tree/PTHR31839
