# Supplementary material for: Dehydration-Responsive Element Binding Protein 1C, 1E, and 1G Promote Stress Tolerance to Chilling, Heat, Drought, and Salt in Rice
Source: Front Plant Sci. 2022 May 24;13:851731. doi: 10.3389/fpls.2022.851731 (PMC9171204; doi:10.3389/fpls.2022.851731)
Supplement: Supplementary file 3 [file Presentation_1.pdf]

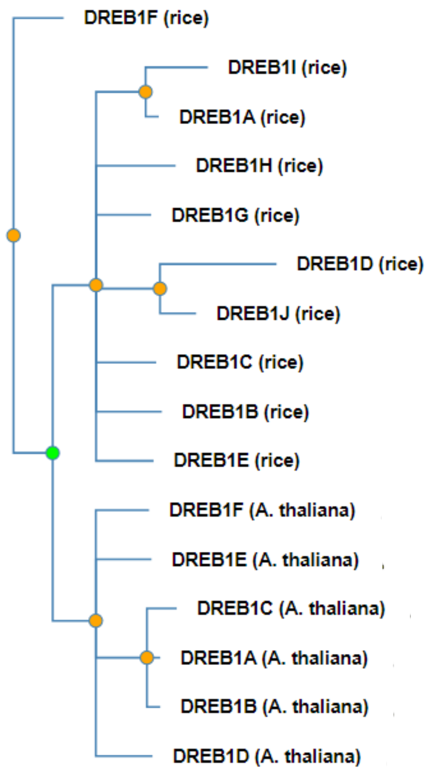

FIGURE S1. Phylogenetic tree of the OsDREB1 and AtDREB1 proteins. The tree is from Phylogenes (http://www.phylogenes.org) using the Arabidopsis *DREB1B* gene as a query on genes from Arabidopsis and rice.

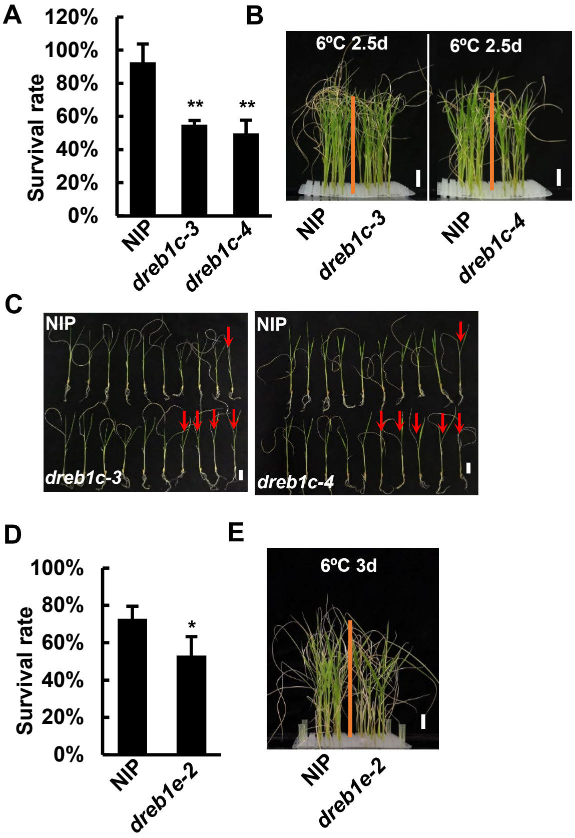

FIGURE S2. Chilling tolerance phenotype of the *dreb1c* and *dreb1e* mutants.

Phenotypes of the *dreb1c-3* and *dreb1c-4* mutants **(A-C)** and the *dreb1e-2* mutant (**D, E**) after 6ºC treatment for 2.5 days (*dreb1c*) or 3 days (*dreb1e*) followed by a recovery growth at 28ºC for a week**.** (**A)** and (**D)** show mean values of survival rates from three biological repeats and error bars indicate standard deviation (SD). Each biological replicate had 24 seedlings. Asterisks indicate significant differences compared to NIP (* $p < 0.05$, ** $p < 0.01$, Student's *t*-test). Bars = 2 cm. (**B**), and (**D**) show phenotypes of seedlings after recovery. (**C**) displays individual seedlings after recovery. Red arrow indicates dead seedlings (no new growth).

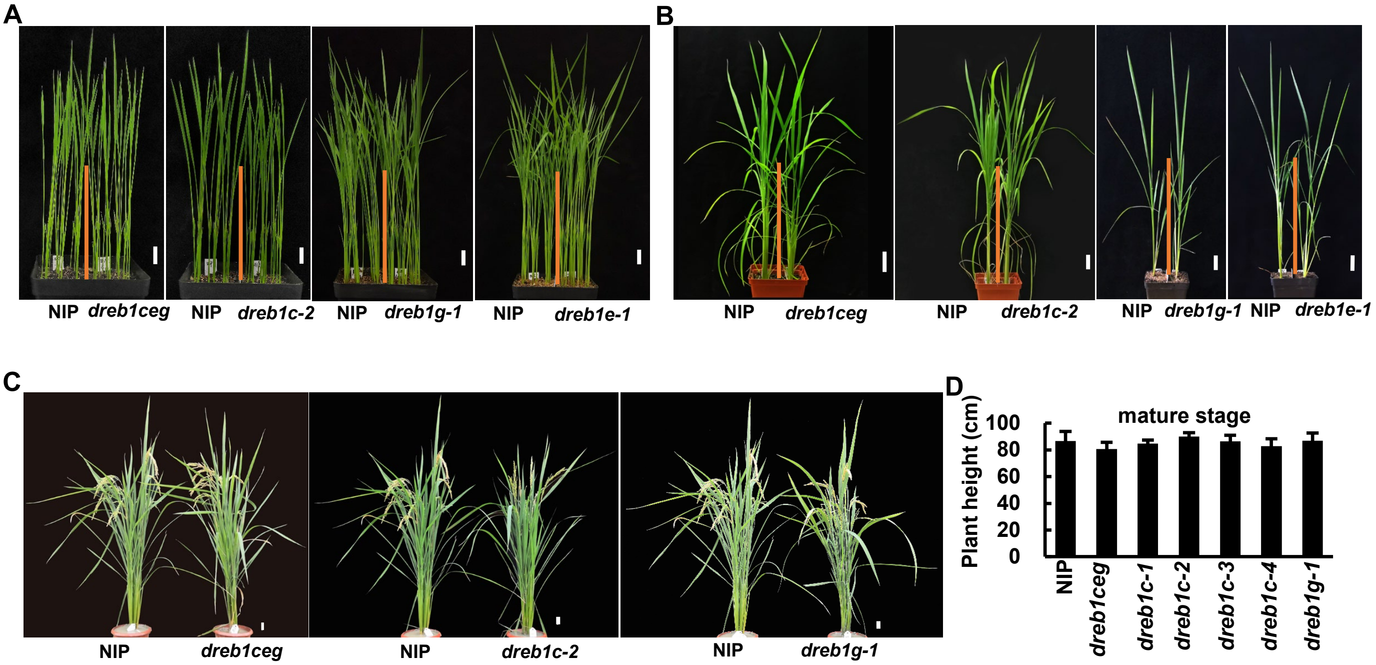

FIGURE S3. The *dreb1ceg* mutants have wild type growth phenotypes under non-stress conditions.

(A-C) Growth phenotypes of NIP and the *dreb1* mutants at seedling stage **(A)**, tillering stage **(B)** and mature stage **(C)**. Bars = 2 cm. **(D)** Plant heights of NIP and the *dreb1* mutants at mature stage. Shown are mean values and error bars are SD.

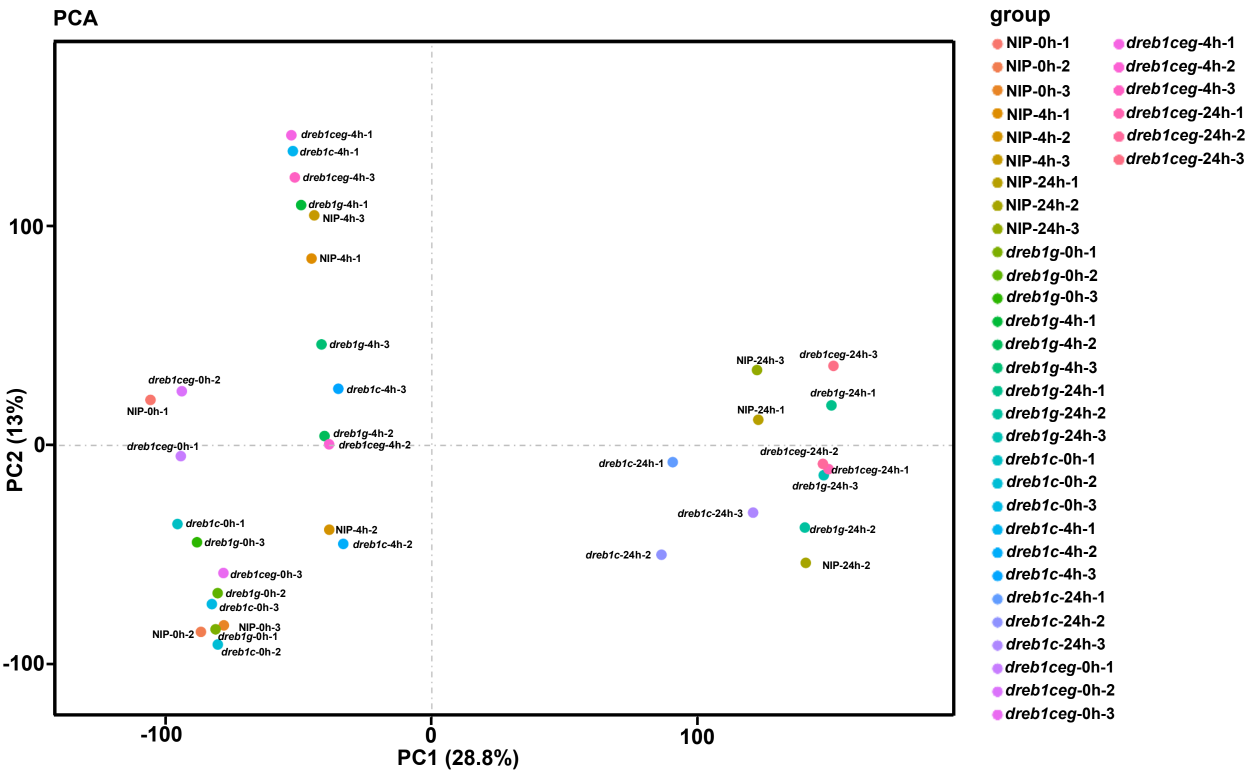

Figure S4. Principal component analysis (PCA) of the transcriptomes of NIP and the *dreb1* mutants. Seedlings of WT NIP, *dreb1c-2*, *dreb1g-1* and *dreb1ceg* were treated at 6ºC for 0 h, 4 h and 24 h. RNA-seq were done on a total of 36 samples (four genotypes, 3 time points and 3 biological replicates). Shown is PC1 and PC2 from PCA analysis (https://www.omicshare.com) on gene expression in these 36 samples.

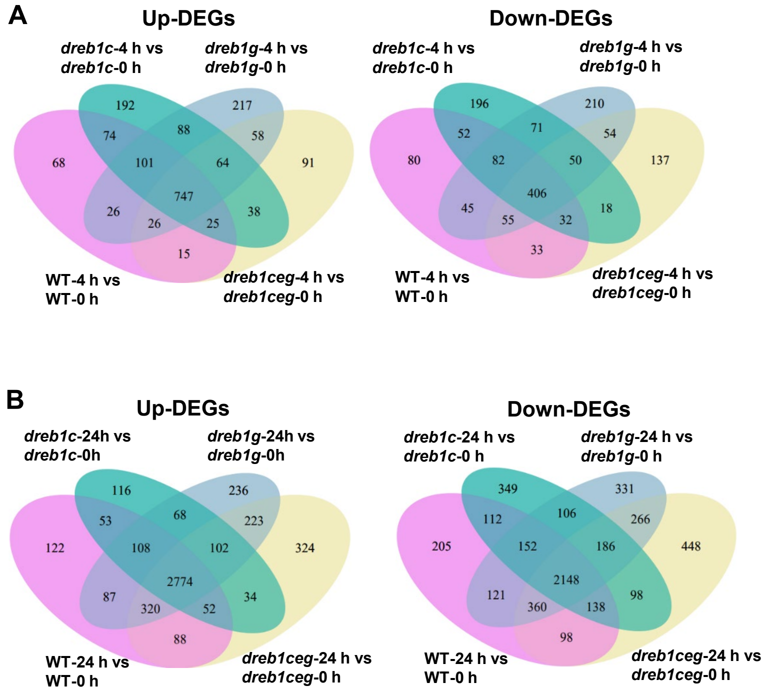

FIGURE S5. Venn diagrams of DEGs responding to chilling stress in the WT and the *dreb1* mutants.

Venn diagrams indicate the overlaps of DEGs in the WT and the *dreb1* mutants at 4 h **(A)** and 24 h **(B)** of 6°C treatment. DEGs are defined by expression changes after chilling versus no chilling with FDR (false discovery rate) ≤ 0.01 and $\log_2$ fold change ≥ 1 (up-DEG) or $\log_2$ fold change ≤ -1 (down-DEG).

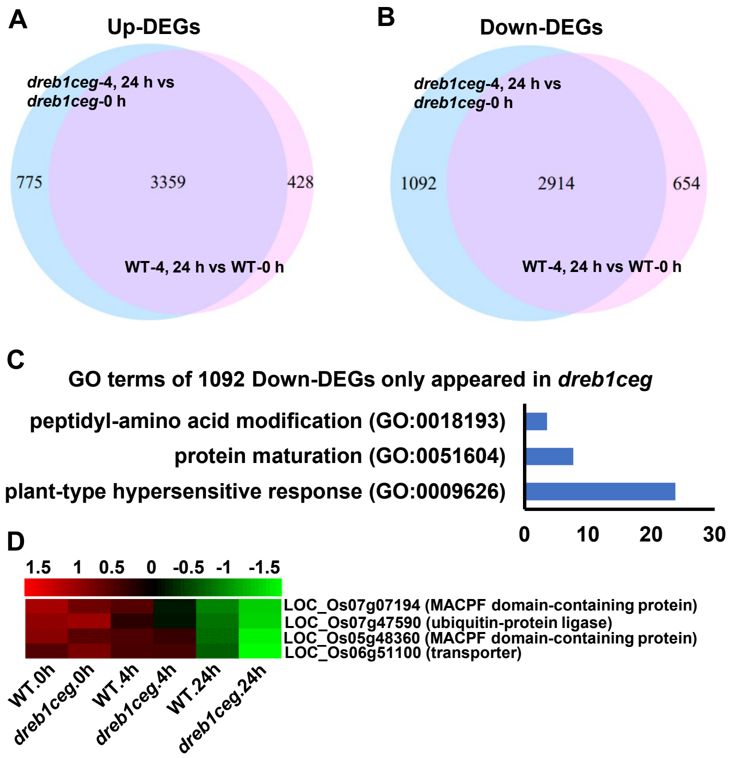

FIGURE S6. GO enrichment analysis of up-DEGs present only in the WT and down-DEGs present only in *dreb1ceg* after chilling treatment.

(A, B) Venn diagrams indicate the numbers of unique and overlapping up-DEGs **(A)** and down-DEGs **(B)** in the WT and the *dreb1ceg* mutant after chilling treatment. **(C)** Enriched GO terms for 1092 down DEGs only appeared in *dreb1ceg*. **(D)** Heat map of expression levels of 4 genes under the ‘plant-type hypersensitive response’ in the WT and *dreb1ceg* at 0 h, 4 h and 24 h of 6°C treatment.

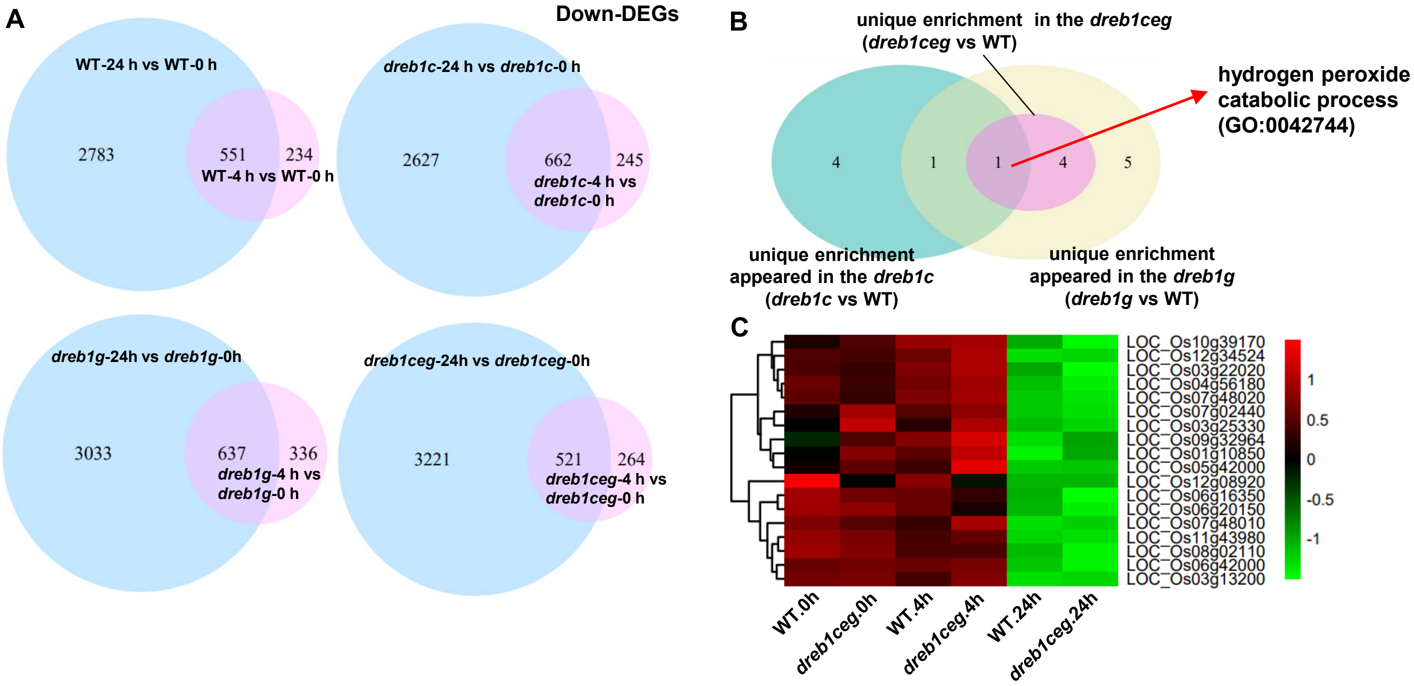

FIGURE S7. GO enrichment analysis of down-DEGs in the WT and the *dreb1* mutants after chilling treatment.

(A) Venn diagrams of down-DEGs of 24 hr 6ºC versus 0 hr and 4 hr 6ºC versus 0 hr in the WT and each of the three *dreb1* mutants. **(B)** Venn diagram of enrichment terms for DEGs of each of the *dreb1* mutants compared to the WT at 6ºC. **(C)** Heat map showing expression levels of 18 peroxidase genes in the WT and the *dreb1ceg* mutant at 0 h, 4 h and 24 h of 6ºC treatment.

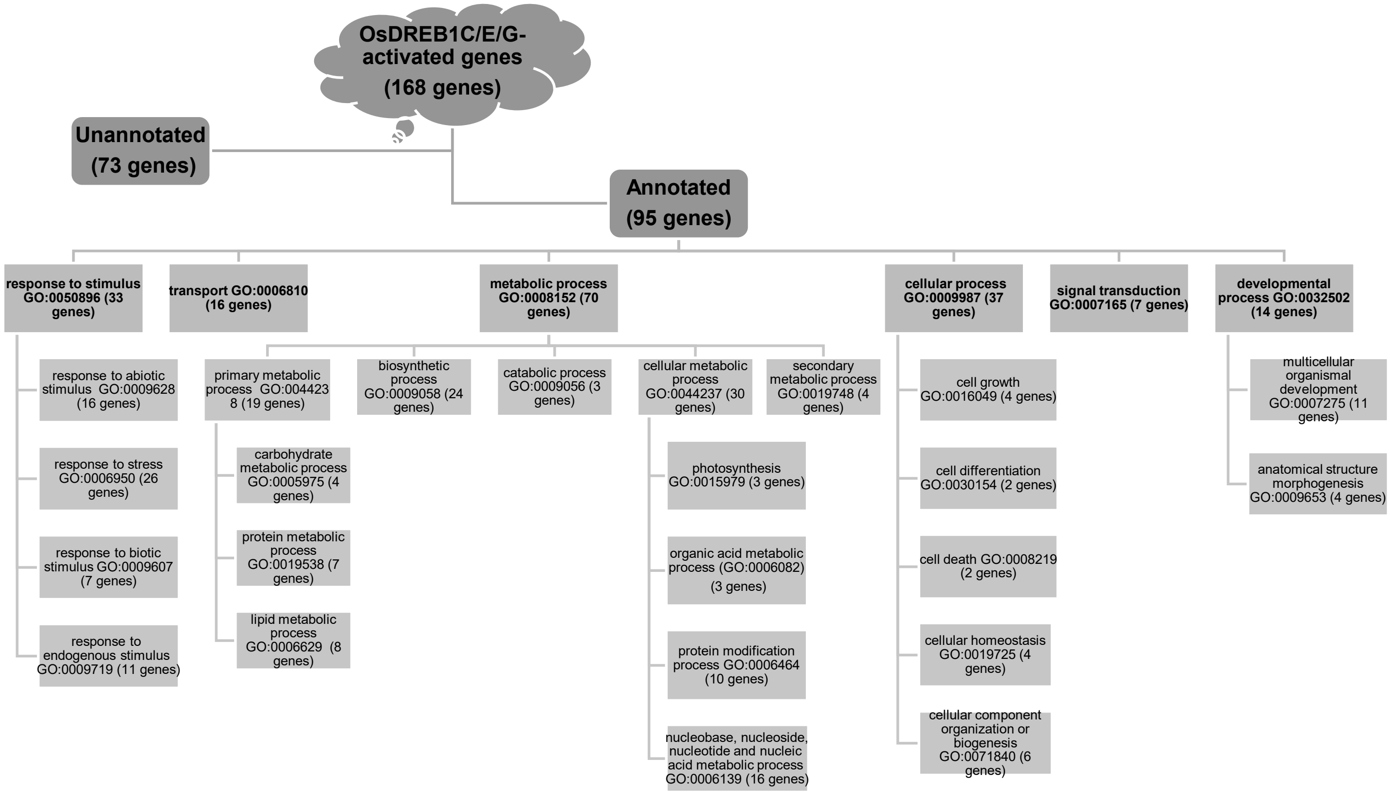

FIGURE S8. Functional classifications of the OsDREB1C/E/G-activated genes according to the gene ontology (GO) annotations of biological process.

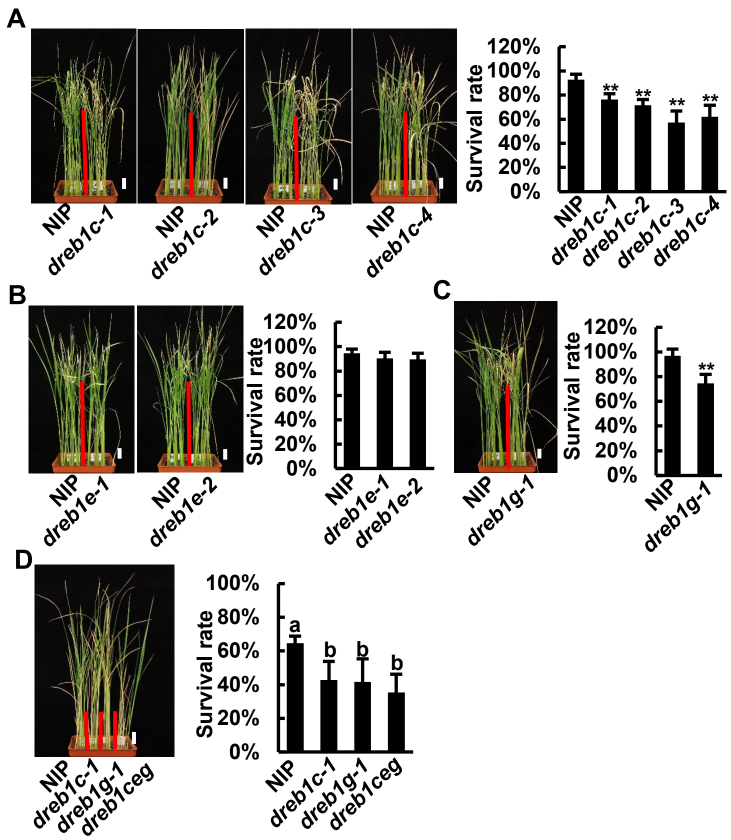

FIGURE S9. The *dreb1c* and *dreb1g* single mutants are more susceptible to salt stress at seedling stage.

Growth phenotypes and survival rates of *dreb1c* **(A)**, *dreb1e* **(B)**, *dreb1g* **(C)** and *dreb1ceg* **(D)** compared with WT NIP, after salt treatment for 23 days (in **A-C**) or 21 days (in **D**). Survival rates are expressed as mean ±SD from three biological replicates each with 21 (in A-C) or 12 (in D) seedlings. Error bar indicates SD. ** indicates significant difference of the mutant from the WT NIP at $p < 0.01$, by student's *t*-test. Lowercase letters above the bars indicate significant differences among samples at p value < 0.05, by SSR-Test. Bars in plant images represent 2 cm.

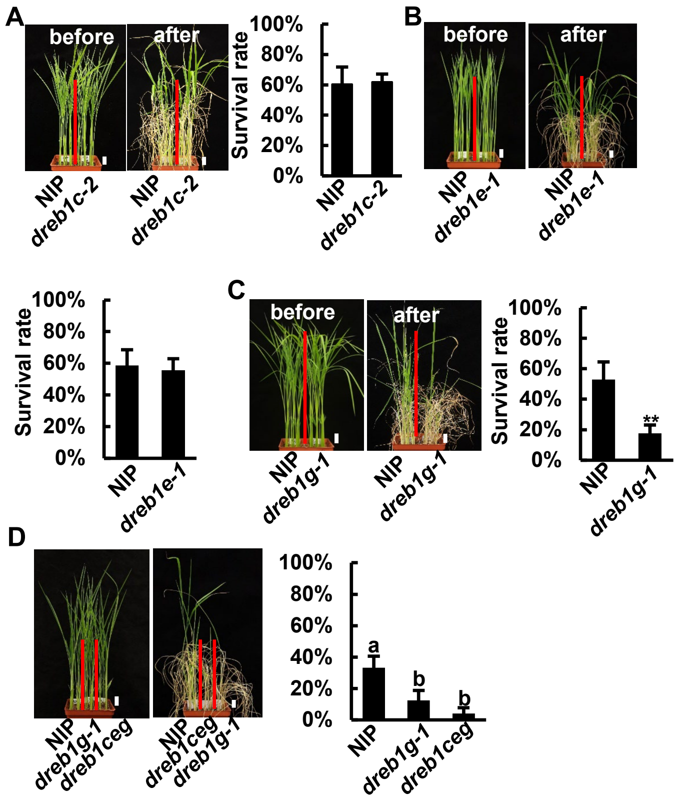

FIGURE S10. The *dreb1g* mutant is more susceptible to drought stress at seedling stage. Growth phenotype and survival rates of *dreb1c* **(A)**, *dreb1e* **(B),** *dreb1g* **(C)** and *dreb1ceg* **(D)** mutants compared with WT NIP with drought treatment. Survival rates are expressed as mean ± SD from three biological replicates each with 21 (in **A-C**) or 16 (in **D**) seedlings. ** indicates a significant difference of the mutant from the WT NIP at $p < 0.01$, by student's t-test. Different letters above the bars indicate significantly different levels among samples at p value < 0.05, by SSR-Test. Bars in plant images represent 2 cm.
